# Identifying Key Genetic Factors Influencing Reproductive Performance in Dairy and Meat Sheep Breeds of the Mediterranean Region

**DOI:** 10.1111/rda.70039

**Published:** 2025-03-17

**Authors:** Maria Consuelo Mura, Giovanni Cosso, Mortadha Ouadday, Chadi Hosri, Joze Starič, Michella Nehme, Vincenzo Carcangiu, Sebastiano Luridiana

**Affiliations:** ^1^ Department of Veterinary Medicine of Sassari University of Sassari Sassari Italy; ^2^ Agris Sardegna Sassari Sassari Italy; ^3^ National School of Veterinary Medicine Sidi Thabet, University of Manouba La Manouba Tunisia; ^4^ Agriculture Lebanese University Dekwaneh Lebanon; ^5^ Clinic for Reproduction and Large Animals—Section for Ruminants University of Ljubljana Ljubljana Slovenia; ^6^ Engineering Holy Spirit University of Kaslik Jounieh Lebanon

**Keywords:** fecundity genes, lambing rate, litter size, Mediterranean sheep, reproductive performances

## Abstract

This study aimed to detect genetic variations in key genes (*BMPR1B*, *BMP15* and *GDF9*) that influence crucial reproductive traits, such as lambing rate and litter size in sheep. Understanding the genetic basis of these traits is essential for developing more efficient breeding strategies and selecting superior breeding stock, ultimately contributing to improved reproductive efficiency across different sheep breeds, production purposes and farming systems. We examined polymorphisms in the above genes across 500 adult ewes from three Mediterranean countries. The studied breeds included high‐yielding dairy (Sarda), dual‐purpose with a meat focus (Awassi) and medium‐to‐low dairy and meat breeds (Slovenian Bovška, Istrian Pramenka and Jezersko‐Solčavska). Genetic variations were analysed and correlated with reproductive efficiency, including the interval in days from ram introduction to lambing based on lambing dates. This was used to assess the potential influence of these genes on follicular maturation and oocyte developmental competence. The analysis identified 15 polymorphisms distributed differently among the breeds, with the Sarda exhibiting 11 SNPs and the Awassi 13. The Slovenian breeds showed distinct genetic variation patterns potentially linked to their production goals. However, no significant association was observed between the identified polymorphisms and the analysed reproductive traits. Further studies on other genes regulating bone morphogenetic protein expression, such as the Gremlin 1 (*GREM1*) gene, may provide additional insights into the genetic mechanisms underlying reproductive traits.

## 1 Introduction

The reproductive performances have an economic impact on the livestock industry. Numerous studies have been conducted over the years to improve reproductive efficiency in sheep farming, as reproduction is a complex and multifaceted system influenced by many environmental factors. Furthermore, several genes are involved in the control of different reproductive traits and have an impact on reproductive performances, including fertility and prolificacy (Drouilhet et al. 2009).

Several major genes, such as bone morphogenetic protein 15 (*BMP15*), growth differentiation factor 9 (*GDF9*) and bone morphogenetic protein receptor 1B (*BMPR1B*), also known collectively as *Fec*‐genes, have been recognised as playing a key role in controlling various reproductive traits in sheep (Drouilhet et al. 2013; Galloway et al. 2000; Hanrahan et al. 2004; Souza et al. 2001). These genes produce specific proteins BMP15, GDF9 and BMPR1B, respectively, with important functions in reproduction and a synergistic effect on litter size in ewes. Exactly, BMP15 and GDF9 are produced by oocytes and bind to the BMPR1B receptor on granulosa cells (Al‐Mutar et al. 2018).

The ovine *BMP15* (also known as *FecX*) gene is located on the X chromosome. It consists of 2 exons and spans the region from 54,283,635 to 54,290,315 on the ARS‐UI_Ramb_v3.0 genome assembly (NCBI RefSeq assembly GCF_016772045.2). Various mutations within the *BMP15* gene sequence have been identified across different sheep breeds, and these are often designated with specific tags. Notably, the *FecXGr* and *FecXO* mutations were found to significantly increase litter size and ovulation rate in homozygous ewes (Demars et al. 2013). Additionally, the SNP rs55628000 was recognised as having a significant effect on ewe litter size (Amini et al. 2018). Interestingly, the *FecXB*, *FecXG*, *FecXL* and *FecXR* mutations displayed improved ovulation rate and increased litter size in heterozygous ewes, while homozygous ewes were completely sterile (Abdoli et al. 2016; Bodin et al. 2007; Galloway et al. 2000; Hanrahan et al. 2004).

*GDF9* (also known as *FecG*) gene is located on chromosome 5 in sheep, ranging from nucleotide 42,113,878 to 42,116,604 of the ARS‐UI_Ramb_v3.0 assembly. It consists of 2 exons and encodes a member of the transforming growth factor‐beta (TGF‐β), which is involved in the development of ovarian follicles and ovulation in sheep (Tang et al. 2018; Wang et al. 2021). Some mutations affecting fertility or sterility have been found in the *GDF9* sequence across different sheep breeds. Specifically, the mutation named *G8* (or *FecG* ^*H*^), at position 42,114,447, has shown addictive effects on prolificacy (Hanrahan et al. 2004). Additionally, the mutation named G1, produced by a G to an A substitution in exon 1 at position 42,116,345, has been found at high frequency in prolific ewes and has been associated with increased ovulation rate and litter size in Middle Eastern and Chilean breeds of sheep (Barzegari et al. 2010; Polley et al. 2010; Paz et al. 2015).

*BMPR1B* locus was the first gene associated with a positive effect on litter size and ovulation rate in Booroola Merino sheep (Mulsant et al. 2001; Smith et al. 1993; Wilson et al. 2001), showing to be a major factor associated with increased ovulation rate in sheep, attributed to the so‐called *Booroola* mutation (or *FecB*). It consists of 16 exons located on chromosome 6, spanning from 30,028,547 to 30,482,585 nucleotides of the ARS‐UI_Ramb_v3.0 assembly.

Due to their role in the ovarian follicle development, these genes are recognised as oocyte‐derived growth factors that are essential for follicular growth beyond the primary stage of development in sheep (Clark et al. 2020). At this point, it can be stated that all these genes can positively influence reproductive performance by promoting follicular maturation and oocyte developmental competence, which in turn can improve ovulation rate and litter size in sheep. Therefore, measuring the interval between the introduction of rams and the subsequent lambing dates may provide insights into the potential influence of these genes on improved follicular development and oocyte maturation.

Improving the understanding of the genes mentioned, even in breeds that have been less studied in this area, could enhance the productivity of local sheep breeds. This would be beneficial, as the production of small ruminants is a crucial source of income in many regions of the Mediterranean basin. So, the aim of this study was to detect genetic variation within some of the important genes affecting litter size in sheep (*BMPR1B*, *BMP15* and *GDF9*) to investigate their effect on reproduction traits in some local dairy and meat breeds of sheep living in different Mediterranean countries.

## 2 Materials and Methods

### 2.1 Animals' Management and Reproductive Data Collection

#### 2.1.1 Sarda Breed

Sarda breed, autochthonous of Sardinia—the second largest island in the Mediterranean Sea—is the most common dairy breed in Italy. Its well‐developed udder with a large and globular cistern and highly implanted teats makes it suitable for both hand and mechanical milking. Due to its small size (42–55 kg), frugality, rusticity and high milk yield (up to 450 L in good breeding conditions), Sarda sheep has spread beyond its region of origin to other areas of the Italian peninsula (Lazio and Tuscany), as well as to other Mediterranean countries like Tunisia and Spain, as the Mediterranean scrub constitutes its ideal habitat. Sardinia has a typical Mediterranean climate, with hot, dry summers and mild, wet winters. Rainfall follows a Mediterranean pattern, with nearly dry summers and rainy autumns, winters and springs. Annual precipitation varies but is generally around 500–700 mm.

This research was conducted on 100 Sarda ewes (3–5 years old, with a body condition score [BCS] ranging from 2.5 to 3.5) from a semiextensive farm in North Sardinia (40.70 N), which raises approximately 800 dairy sheep of the Sarda breed. The enrolled animals grazed on leguminous and gramineous grasses during the day and were penned at night with free access to hay (11.1% raw protein and 7.2 MJ ME/kg DM) and water. They also received 400 g of commercial concentrated food per head (20.4% of raw protein and 12.5 MJ ME/kg of DM) twice a day, during the two daily milkings. Five fertile adult rams (male/female ratio: 1/20) were introduced on 15 May and removed from the group after 50 days. Lambing dates were collected from 150 to 210 days after the rams were introduced. Subsequently, lambing rate (the ratio between the number of ewes that lambed, and the ewes exposed to the rams) and litter size (number of newborn lambs per ewe that lambed) were calculated. Furthermore, the distance, measured in days, from the introduction of rams into the flock and the lambing dates of the ewes (DRIL) was assessed to evaluate the potential influence of these genes on enhanced follicular development and oocyte maturation.

#### 2.1.2 Lebanese Awassi Breed

Awassi sheep is a fat‐tailed sheep that is widespread in the Middle East and Mediterranean basin, and the only sheep breed reared in Lebanon and Palestine. It was also found in the desert of Saudi Arabia, where the term ‘Awassi’ means ‘sheep’ in Arabic. There are many variants of the Awassi breed subpopulation, depending on rearing areas and farming systems, but they are generally well suited to harsh environments due to their desirable features such as endurance to nutritional fluctuations and high environmental temperatures (Hosri et al. 2016; Said et al. 1999). In Lebanon, Awassi sheep is a dual‐purpose breed, with a dominance of meat production, mainly reared by small farmers in marginal areas (Hosri et al. 2016). Although twin or triplet births are sometimes recorded in Awassi ewes, their low general litter size restricts the use of this breed in extensive or semiextensive farming systems, where the poor availability of natural vegetation is the rate‐limiting factor for lamb and milk production (Gootwine et al. 2008).

The present research was conducted on 100 Awassi ewes from two semiintensive farms located in the Bekaa and Jezzine regions, situated in the North‐East and South of Lebanon respectively. The animals were managed in accordance with the recommendations of regulatory agencies responsible for overseeing animal welfare and health across various jurisdictions. Blood samples were collected by the National Health Veterinary Service during routine flock health control procedures. The Bekaa region is a valley situated approximately 30 km east of Beirut, nestled between the Mount Lebanon range to the west and the Anti‐Lebanon Mountains to the east. It is part of the larger Great Rift Valley system, which extends from Syria to the Red Sea, with an average elevation of around 750 m above sea level. The region experiences limited rainfall and has an average annual temperature of 18°C.

The other region, Jezzine, is situated 40 km south of Beirut, surrounded by mountain peaks and pine forests. It has an average altitude of 800 m above sea level and typically experiences an annual temperature of 15°C.

In both regions, the surrounding mountains create a rain shadow that limits precipitation coming from the sea, with a certain degree of increase during the winter, especially from November to February, whereas the summer is very hot and characterised by long dry periods. On both farms, animals were housed during the night and released to graze in nearby pastures during the day after the morning milking was performed. In addition to the grass received from grazing, the animals were fed a daily cereal‐based supplement consisting of yellow corn, wheat and barley (11.71% of crude protein and 14.12 MJ ME/kg DM) upon returning to the fold. Before collecting research data, all the animals were examined by a veterinarian to assess their good health. Subsequently, they were enrolled for the trial based on sex (only female), age (ranging 3–6 years) and body condition score (BCS, mean 3.5 ± 0.5). The ewes, all multiparous, were exposed to the rams from July 10th to September 10th with a male/female ratio of 1/20, and a total of five rams, to obtain lambing periods from the middle of December to the end of January. Lambing dates were collected from 150 to 210 days after ram introduction, during which lambing rate (number of ewes that lambed per ewe exposed to the ram), litter size (number of newborn lambs per ewes that lambed) and the DRIL were recorded.

#### 2.1.3 Slovenian Breeds

A total of 300 adult ewes of three Slovenian local breeds—Bovška (*n* = 100), Istrian Pramenka (*n* = 100) and Jezersko‐Solčavska (*n* = 100) were used for the present research.

The Bovška is a small‐to‐medium‐sized breed (40–50 kg average adult weight) raised in extensive farming systems primarily for milk production (mean milk production level of 150 kg in 6 months of lactation). Ewes typically lamb once per year (rams introduction in the flock in October for a 60‐day period, with lambing season in March–April) with an average breed litter size of approximately 1.2 lambs per parturition.

The Bovška ewes were raised near the town of Bovec in the north‐western region of Slovenia, a few kilometres from the Italian–Slovenian border. The valley is surrounded by high mountains where vegetation grows lush; the climate is more continental than Mediterranean in nature. Winters are cold, although not harsh, and summers bring hot and high temperatures. The animals were penned indoors at night with free access to hay and water and grazed in a mixture of grass and legume pastures during the day. Additionally, they received a supplement of 200 g of corn grain during the twice‐daily milking session.

The Istrian Pramenka is a dual‐purpose (milk and meat) breed with a dominance of milk production, with an average milk yield of 195 kg in 7 months of lactation. It is a medium‐large sized breed, weighing between 75 and 95 kg and has an average litter size of 1.13 live lambs per lambing. The ewes usually lamb in February–March (rams are typically introduced in September for a 60‐day period) and are managed and fed in a similar manner to the Bovška breed. The enrolled ewes were raised near the Vremščica mountain, one of three peaks on the Karst plateau that exceed 1,000 m above sea level; the vast and relatively gently sloping meadows provide luxurious grazing to sheep.

The Jezersko‐Solčavska is a larger breed compared to the other two, weighing 85–100 kg, and it is used for both meat and wool production. Ewes of this breed can lamb twice a year as they are fertile throughout the year and quite frequently have twin lambs (average litter size of 1.4). The animals were managed in extensive conditions, with feed supplementation provided only when adverse climatic conditions made it strictly necessary to integrate pasture feeding. Rams were joined with ewes all the year round.

The male‐to‐female ratio was 1/20 across all three breeds, with five rams for every 100 ewes. Consequently, a total of 15 rams were introduced for the 300 enrolled Slovenian ewes. All the enrolled ewes were between 3 and 7 years old. The reproductive parameters recorded were lambing date, number of ewes that lambed per ewe exposed to the ram (lambing rate), number of newborn lambs per ewe that lambed (litter size) and distance, in days, between the introduction of males into the flock and the lambing dates of the ewes (DRIL). For the Jezersko‐Solčavska breed, all the lambing events in a whole year from June to June were recorded.

### 2.2 Blood Sampling and Genotyping

The farms enrolled were assisted by the official veterinary service of their country. This study used blood provided by Veterinarians of the National Health Service who took blood samples as part of the standard processes for flock health management. Accordingly, no further ethics statement was needed for the present study. Genomic DNA was extracted from 200 μL of whole blood, collected by means of a sterile vacuum tube with ethylenediaminetetraacetic acid as an anticoagulant, using a commercial extraction kit (Genomic DNA from blood, Macherey—Nagel, Düren, Germany). The concentration and purity of the samples were evaluated by spectrophotometric readings before the analysis using Eppendorf BioPhotometer (Eppendorf AG, Hamburg, Germany). Approximately 100–150 ng of genomic DNA was subjected to polymerase chain reaction (PCR), using MAXYGENE II Thermal Cycler (Axygen Tewksbury, MA, USA). Primers used for the PCR amplification of *BMP15* gene exons 1 and 2 (producing F1 and F2 fragments respectively), of *GDF9* gene exons 1 and 2 (fragments F3 and F4 respectively) and of *BMPR1B* gene exon 8 (F5) are reported in Table 1. It should be noted that the primers for F5 were designed based on Talebi et al. (2018), who referred to this region as exon 7. However, more recent sequence alignment confirms that the amplified region corresponds to exon 8, and this fragment is therefore referred to as exon 8 in the present study. All the fragments were amplified in a reaction mix containing 2.5 μL of template DNA, 5 μL of 10x PCR buffer (50% glycerol (v/v), 20 mM Tris–HCl (pH 8.7), 100 mM KCl, 0.1 mM EDTA, stabilisers), 3 μL and 4 μL of 25 mM MgCl_2_ for fragments F1–F4 and for F5, respectively, 10 pM of each primer, 8 μL of 1.25 mM of each dNTPs and 0.25 μL of 5 U/μl Taq DNA polymerase (HOTFIREPol DNA Polymerase Solis BioDyne, Teaduspargi, Tartu, Estonia). The PCR reaction conditions were according to previous literature: Amini et al. (2018) for F1, Calvo et al. (2020) for F2, Abdoli et al. (2018) for F3 and F4 and Talebi et al. (2018) for F5. Electrophoresis of PCR products was performed in a 1% TAE buffer on a 1.5% (w/v) agarose gel (GellyPhor, Eu‐roclone, UK) containing 9 μL of RedSafe Dna Stain 20,000X (iNtROn Biotechnology) in parallel with a 100 bp marker (Solis BioDyne, Teaduspargi, Tartu, Estonia) at a constant voltage of 100 V for 30 min and then visualised using a UV light trans‐illuminator (UVItec, Cambridge, UK). The PCR products were purified and sequenced in both directions by a commercial sequencing service (BioFab Research srl, Roma, Italy) using Applied Biosystems 3730 DNA Analyser (Perkin‐Elmer Applied Biosystems, Foster City, CA, USA). The Basic Local Alignment Search Tool (BLAST, https://blast.ncbi.nlm.nih.gov/Blast.cgi), a resource developed by the National Centre for Biotechnology Information (NCBI) was used to compare the obtained sequences with the genome version ARS‐UI_Ramb _v3.0 (GenBank assembly accession number: GCF_016772045.2). Nucleotide sequence alignments and comparisons were carried out using the BioEdit Sequence Alignment Editor software (http://www.mbio.ncsu.edu/BioEdit/BioEdit.html).

**TABLE 1:** PCR ID fragments, genes and region of amplification, primers sequences, PCR product size in base pair (bp) and references.

ID	Gene	Region	Primer sequences (5′–3′)	Product size	Reference
F1	BMP15	Exon 1	Fw: TCCTTGCCCTATCCTTTGTG	482 bp	Amini et al. 2018
			Rv: CCCTCCCACCAGAACAATA		
F2		Exon 2	Fw: CATCTCAAGGCTGCTTGTCA	1006 bp	Calvo et al. 2020
			Rv: TTCGAATTTCTTGGGCAAAC		
F3	GDF9	Exon 1	Fw: GAAGACTGGTATGGGGAAATG	462 bp	Abdoli et al. 2018
			Rv: CCAATCTGCTCCTACACACCT		
F4		Exon 2	Fw: TGGCATTACTGTTGGATTGTTT	1019 bp	Abdoli et al. 2018
			Rv: GGTTTTACTTGACAGGAGTCTG		
F5	BMPR1B	Exon 8	Fw: AGTGTGTTGGGGGATTTA	269 bp	Talebi et al. 2018
			Rv: AAAGAGAGGAAAGCTAGGAA		

### 2.3 Statistical Analysis

R statistical software (Version 4.3.2 R Core Team 2023 R: A language and environment for statistical computing. R Foundation for Statistical Computing, Vienna, Austria. URL https://www.r‐project.org/) was used for analysing the data. The Hardy–Weinberg equilibrium of each genotype was evaluated using the Hardy–Weinberg Rpackage (Graffelman 2015). To assess for normal distribution and homogeneity of variances of litter size, the Shapiro–Wilk and Levene tests were used respectively. Subsequently, the association between each genotype and litter size and DRIL was estimated using the following linear model:

$$Y_{\mathrm{jkl}}=\mu +G_{j}+R_{k}+e_{\mathrm{jkl}}$$

Where Y_jkl_ is the phenotypic value of litter size, or DRIL, μ is the overall mean, G_j_ is the fixed effect of the genotype (three levels), R_k_ is the random effect of the ram (5 levels) and e_jkl_ is the random residual effect of each observation. The model was fitted separately for each breed to account for potential breed‐specific differences in genetic and phenotypic variability. At first, the model included the farm and age effects, but once established they were not significant, they were removed by the model. The differences among genotypes in lambing rate were analysed using a Chi‐square test. All the values were considered significant at *p* < 0.05, and the results of the model are shown as least square means ± standard error means (LSmean ± SEM).

## 3 Results

For all the studied breeds, the fragments obtained by the PCR reactions were of the expected size (Table 1). A total of 15 polymorphisms were found in the analysed exons, distributed differently across the studied breeds. The Sarda ewes exhibited a total of 11 single nucleotide variations (SNPs); 13 were found in Awassi. The Slovenian breeds showed a varied trend in genetic variation, potentially linked to their different production purposes. A summary table (Table 2) illustrates the variability found in the breeds taken into consideration for a quicker comparison, while the details of the variations are visible in Tables 3, 4, 5, for the Sarda, the Awassi and the Slovenian breeds respectively. All variations were mapped to the ARS‐UI_Ramb _v3.0 sheep genome assembly version, with RefSeq assembly accession GCA_016772045.2. Four SNPs caused amino acid changes and were analysed using the Ensembl Variant Effect Predictor (VEP) to evaluate their SIFT (Sorting Intolerant From Tolerant) and, consequently, to predict whether the amino acid substitutions affect protein function. In the *BMP15* gene, the variation ID rs592773279, present in all the examined breeds, was characterised by a deletion of a leucine codon at position 11 of the protein sequence (NP_001108239). All the amino acid substitutions were predicted to be tolerated except for the SNP rs589708049 found only in the Istrian Pramenka breed, which recorded a deleterious SIFT, as reported in Table 6. The Hardy–Weinberg equilibrium (HWE) was calculated and reported for all the found variations in each of the studied breeds, as shown in Tables 3, 4, 5. The enrolled populations generally exhibited Hardy–Weinberg equilibrium, with some exceptions observed in specific breeds. In the Sarda ewes, the SNPs rs592773279 and rs422644056 showed deviations from Hardy–Weinberg equilibrium due to an excess of homozygotes, while rs160076413 exhibited an excess of heterozygotes. The Awassi breed displayed deviations in SNPs rs587958054 and rs160076413, both due to an excess of homozygotes. The Slovenian breeds presented the most diverse patterns, with deviations from Hardy–Weinberg equilibrium observed in several SNPs: rs592773279 showed an excess of homozygous ewes in the Bovška and Istrian Pramenka breeds; rs160076413 was in disequilibrium across all Slovenian breeds (excess of homozygotes); rs160076408 in the Istrian Pramenka breed (excess of homozygotes); rs193637058, rs399579080 and rs421019907 exhibited an excess of homozygous ewes in the Bovška breed; rs589708049 in the Istrian Pramenka breed had an excess of homozygotes; rs408447622 and rs427897187 showed an excess of homozygotes in the Bovška ewes; and rs409518279 in the Jezersko‐Solčavska breed had an excess of heterozygous ewes.

**TABLE 2:** A summary of the genes, chromosomes (Chr), variation positions and variation IDs found in the studied sheep breeds according to the ARS‐UI_Ramb_v3.0 reference genome.

Gene	Chr	Variation position	Variation ID	Exon	Breeds				
					Sarda	Awassi	Slovenian		
							J	B	P
*BMP15*	X	54,290,286–8	rs592773279	1	✓	✓	✓	✓	✓
		54,284,258	rs55628000	2	—	✓	—	—	—
		54,283,966	rs587958054	2	—	✓	—	—	—
*GDF9*	5	42,116,345	rs410123449	1	✓	✓	✓	—	—
		42,115,016	rs422644056	2	✓	✓	✓	✓	—
		42,115,010	rs160076413	2	✓	✓	✓	✓	✓
		42,114,899	rs1087316417	2	—	—	—	—	✓
		42,114,776	rs160076408	2	✓	✓	✓	✓	✓
		42,114,737	rs193637058	2	✓	✓	✓	✓	✓
		42,114,509	rs399579080	2	✓	✓	✓	✓	✓
		42,114,493	rs421019907	2	✓	✓	✓	✓	✓
		42,114,447	rs589708049	2	—	—	✓	✓	✓
*BMPR1B*	6	30,050,773	rs408447622	8	✓	✓	✓	✓	✓
		30,050,770	rs427897187	8	✓	✓	✓	✓	✓
		30,050,567	rs409518279	8	✓	✓	✓	✓	✓

Abbreviations: B, Bovška; J, Jezersko‐Solčavska; P, Istrian Pramenka.

**TABLE 3:** Genes, chromosome (Chr), variation position and ID according to ARS‐UI_Ramb_v3.0, exon, genotypes, alleles and their frequency (expressed as percentage) and *p*‐value for Hardy–Weinberg equilibrium in the studied Sarda breed ewes (*n* = 100).

Gene	Chr	Variation ID	Exon	Genotype	Genotype frequency	Allele	Allele frequency	HW
*BMP15*	X	rs592773279	1	AAG/AAG	0.45	AAG	0.56	0.000
				AAG/del	0.22	del	0.44	
				del/del	0.33			
*GDF9*	5	rs410123449	1	CC	0.65	C	0.81	0.692
				CT	0.32	T	0.19	
				TT	0.03			
		rs422644056	2	GG	0.83	G	0.90	0.002
				GA	0.13	A	0.10	
				AA	0.04			
		rs160076413	2	CC	0.02	C	0.46	0.000
				CT	0.87	T	0.54	
				TT	0.11			
		rs160076408	2	CC	0.65	C	0.81	0.900
				CT	0.31	T	0.19	
				TT	0.04			
		rs193637058	2	CC	0.83	C	0.92	0.353
				CT	0.17	T	0.08	
				TT	0.00			
		rs399579080	2	TT	0.93	T	0.97	0.717
				TC	0.07	C	0.03	
				CC	0.00			
		rs421019907	2	CC	0.93	C	0.97	0.717
				CT	0.07	T	0.03	
				TT	0.00			
*BMPR1B*	6	rs408447622	8	CC	0.69	C	0.82	0.087
				CT	0.25	T	0.18	
				TT	0.06			
		rs427897187	8	CC	0.32	C	0.59	0.338
				CT	0.53	T	0.41	
				TT	0.15			
		rs409518279	8	GG	0.75	G	0.86	0.126
				GA	0.21	A	0.14	
				AA	0.04			

**TABLE 4:** Genes, chromosome (Chr), variation ID according to ARS‐UI_Ramb_v3.0, exon, genotypes, alleles and their frequency (expressed as percentage) and *p* value for Hardy–Weinberg equilibrium in the studied Awassi ewes (*n* = 100).

Gene	Chr	Variation ID	Exon	Genotype	Genotype frequency	Allele	Allele frequency	HW
*BMP15*	X	rs592773279	1	AAG/AAG	0.60	AAG	0.76	0.219
				AAG/del	0.32	del	0.24	
				del/del	0.08			
		rs55628000	2	AA	0.66	A	0.80	0.086
				AG	0.27	G	0.20	
				GG	0.07			
		rs587958054	2	CC	0.81	C	0.84	0.000
				CT	0.06	T	0.16	
				TT	0.13			
*GDF9*	5	rs410123449	1	CC	0.73	C	0.84	0.069
				CT	0.22	T	0.16	
				TT	0.05			
		rs422644056	2	GG	0.70	G	0.82	0.061
				GA	0.24	A	0.18	
				AA	0.06			
		rs160076413	2	CC	0.84	C	0.87	0.000
				CT	0.06	T	0.13	
				TT	0.10			
		rs160076408	2	CC	0.66	C	0.80	0.086
				CT	0.27	T	0.20	
				TT	0.07			
		rs193637058	2	CC	0.70	C	0.82	0.061
				CT	0.24	T	0.18	
				TT	0.06			
		rs399579080	2	TT	0.64	T	0.78	0.066
				TC	0.28	C	0.22	
				CC	0.08			
		rs421019907	2	CC	0.68	C	0.81	0.120
				CT	0.26	T	0.19	
				TT	0.06			
*BMPR1B*	6	rs408447622	8	CC	0.66	C	0.80	0.086
				CT	0.27	T	0.20	
				TT	0.07			
		rs427897187	8	CC	0.31	C	0.58	0.278
				CT	0.54	T	0.42	
				TT	0.15			
		rs409518279	8	GG	0.73	G	0.84	0.069
				GA	0.22	A	0.16	
				AA	0.05			

**TABLE 5:** Genes, chromosome (Chr), variation position and ID according to ARS‐UI_Ramb_v3.0, exons (Ex), genotypes, alleles and their frequency (expressed as percentage) and *p* value for Hardy–Weinberg equilibrium in the studied Slovenian breed ewes (J = Jezersko‐Solčavska, *n* = 100; B = Bovška, *n* = 100 and P = Istrian Pramenka, *n* = 100).

Gene	Chr	Variation ID	Exon	Genotype	Genotype frequency			Allele	Allele frequency			HW		
					J	B	P		J	B	P	J	B	P
*BMP15*	X	rs592773279	1	AAG/AAG	0.48	0.71	0.50	AAG	0.69	0.71	0.59	0.886	0.000	0.000
				AAG/del	0.43	0.00	0.17	del	0.31	0.29	0.41			
				del/del	0.09	0.29	0.33							
*GDF9*	5	rs410123449	1	CC	0.75	—	—	C	0.86	—	—	0.126	—	—
				CT	0.21	—	—	T	0.14	—	—			
				TT	0.04	—	—							
		rs422644056	2	GG	0.75	0.62	—	G	0.86	0.81	—	0.126	0.019	—
				GA	0.21	0.38	—	A	0.14	0.19	—			
				AA	0.04	0.00	—							
		rs160076413	2	CC	0.29	0.24	0.46	C	0.48	0.42	0.63	0.010	0.005	0.003
				CT	0.37	0.35	0.33	T	0.52	0.58	0.37			
				TT	0.34	0.41	0.21							
		rs1087316417	2	TT	—	—	0.92	T	—	—	0.96	—	—	0.677
				TA	—	—	0.08	A	—	—	0.04			
				AA	—	—	0.00							
		rs160076408	2	CC	0.75	0.95	0.79	C	0.86	0.98	0.88	0.126	0.798	0.026
				CT	0.21	0.05	0.17	T	0.14	0.02	0.12			
				TT	0.04	0.00	0.04							
		rs193637058	2	CC	0.67	0.73	0.83	C	0.82	0.82	0.92	0.702	0.000	0.353
				CT	0.29	0.17	0.17	T	0.18	0.18	0.08			
				TT	0.04	0.10	0.00							
		rs399579080	2	TT	0.71	0.88	0.83	T	0.84	0.91	0.92	0.354	0.000	0.353
				TC	0.25	0.06	0.17	C	0.16	0.09	0.08			
				CC	0.04	0.06	0.00							
		rs421019907	2	CC	0.71	0.88	0.83	C	0.84	0.91	0.92	0.354	0.000	0.353
				CT	0.25	0.06	0.17	T	0.16	0.09	0.08			
				TT	0.04	0.06	0.00							
		rs589708049	2	AA	—	0.74	0.79	A	—	0.81	0.88	—	0.000	0.026
				GA	—	0.14	0.17	G	—	0.19	0.12			
				GG	—	0.12	0.04							
*BMPR1B*	6	rs408447622	8	CC	0.13	0.79	0.88	C	0.42	0.82	0.94	0.057	0.000	0.523
				CT	0.58	0.05	0.12	T	0.58	0.18	0.06			
				TT	0.29	0.16	0.00							
		rs427897187	8	CC	0.96	0.74	0.33	C	0.98	0.85	0.56	0.838	0.047	0.506
				CT	0.04	0.21	0.46	T	0.02	0.15	0.44			
				TT	0.00	0.05	0.21							
		rs409518279	8	GG	0.54	0.70	0.96	G	0.77	0.82	0.98	0.003	0.061	0.838
				GA	0.46	0.24	0.04	A	0.23	0.18	0.02			
				AA	0.00	0.06	0.00							

**TABLE 6:** Genes, variation ID, amino acid (aa) change, SIFT (sorting intolerant from tolerant) and *p*‐value for Hardy–Weinberg equilibrium in the studied breeds of sheep: Sarda (*n* = 100), Awassi (*n* = 100) and Slovenian (*n* = 300).

Gene	Variation ID	aa change	SIFT	HW
*BMP15*	rs592773279	deletion 11 Leu		0.219
	rs55628000	Leu 261 Pro	0.16	0.086
	rs587958054	—		0.000
*GDF9*	rs410123449	Arg 87 His	0.18	0.069
	rs422644056	—		0.061
	rs160076413	—		0.000
	rs1087316417	—		—
	rs160076408	Glu 241 Lys	0.7	0.086
	rs193637058	—		0.061
	rs399579080	—		0.066
	rs421019907	Val 332 Ile	0.19	0.120
	rs589708049	Phe 395 Ser	0.02	—
*BMPR1B*	rs408447622	—		0.086
	rs427897187	—		0.278
	rs409518279	—		0.069

Although numerous mutations were found across the different breeds, statistical analysis did not reveal any significant association between the polymorphisms in the three genes analysed and lambing rate or litter size, as shown in Table 7. Additionally, the distance, measured in days, between the introduction of rams into the flock and the subsequent lambing dates (DRIL), did not show any statistically significant differences among the observed breeds (Table 8).

**TABLE 7:** Gene, SNPs ID, genotypes, lambing rate (%) and litter size (LSmeans ± SEM), in the studied breeds of sheep: Awassi (*n* = 100), Sarda (*n* = 100) and Slovenian (J = Jezersko‐Solčavska, *n* = 100; B = Bovška, *n* = 100 and P = Istrian Pramenka, *n* = 100).

			Sarda breed	Awassi breed			Slovenian breeds					
							J	B	P	J	B	P
Gene	SNP ID	Genotype	Lambing rate	Litter size	Lambing rate	Litter size	Lambing rate			Litter size		
*BMP15*	rs592773279	AAG	89.0	1.2 ± 0.1	88.3	1.5 ± 0.1	88.4	87.2	86.8	1.5 ± 0.1	1.2 ± 0.1	1.0 ± 0.1
		AAG/del	88.1	1.1 ± 0.1	85.7	1.4 ± 0.2	86.6	87.9	87.5	1.4 ± 0.2	1.1 ± 0.1	1.1 ± 0.1
		del	88.2	1.1 ± 0.2	87.0	1.5 ± 0.1	87.3	88.2	87.1	1.5 ± 0.1	1.2 ± 0.2	1.0 ± 0.2
	rs55628000	AA	—	—	88.3	1.4 ± 0.1	—	—	—	—	—	—
		AG	—	—	86.5	1.4 ± 0.2	—	—	—	—	—	—
		GG	—	—	86.2	1.4 ± 0.2	—	—	—	—	—	—
	rs587958054	CC	—	—	86.3	1.4 ± 0.2	—	—	—	—	—	—
		CT	—	—	87.4	1.5 ± 0.1	—	—	—	—	—	—
		TT	—	—	87.3	1.5 ± 0.1	—	—	—	—	—	—
*GDF9*	rs410123449	CC	88.1	1.1 ± 0.1	87.8	1.5 ± 0.1	88.2	—	—	1.5 ± 0.1	—	—
		CT	88.4	1.1 ± 0.2	87.0	1.5 ± 0.1	87.1	—	—	1.4 ± 0.2	—	—
		TT	88.6	1.2 ± 0.1	86.2	1.4 ± 0.2	87.3	—	—	1.4 ± 0.1	—	—
	rs422644056	GG	89.6	1.2 ± 0.1	86.8	1.4 ± 0.2	87.2	87.7	—	1.5 ± 0.1	1.1 ± 0.1	—
		GA	88.0	1.1 ± 0.2	88.0	1.5 ± 0.1	87.3	87.7	—	1.4 ± 0.2	1.1 ± 0.1	—
		AA	87.5	1.2 ± 0.1	86.2	1.4 ± 0.2	87.9	88.0	—	1.5 ± 0.1	1.2 ± 0.1	—
	rs160076413	CC	88.4	1.2 ± 0.1	87.6	1.5 ± 0.1	88.4	88.2	86.9	1.5 ± 0.1	1.1 ± 0.1	1.0 ± 0.2
		CT	87.8	1.1 ± 0.1	86.6	1.4 ± 0.2	87.1	87.6	86.9	1.4 ± 0.2	1.1 ± 0.2	1.1 ± 0.1
		TT	88.9	1.2 ± 0.2	86.8	1.4 ± 0.2	87.1	87.5	87.4	1.5 ± 0.1	1.2 ± 0.1	1.0 ± 0.1
	rs108736417	TT	—	—	—	—	—	—	86.7	—	—	1.0 ± 0.1
		TA	—	—	—	—	—	—	87.1	—	—	1.0 ± 0.2
		AA	—	—	—	—	—	—	87.4	—	—	1.0 ± 0.1
	rs160076408	CC	88.9	1.2 ± 0.1	86.2	1.4 ± 0.2	86.4	87.2	88.0	1.4 ± 0.1	1.2 ± 0.1	1.0 ± 0.1
		CT	87.4	1.2 ± 0.2	88.1	1.5 ± 0.1	87.6	87.6	86.8	1.5 ± 0.2	1.1 ± 0.2	1.1 ± 0.2
		TT	88.8	1.2 ± 0.1	86.7	1.4 ± 0.2	88.4	88.5	86.4	1.5 ± 0.2	1.2 ± 0.1	1.1 ± 0.2
	rs193637058	CC	89.0	1.2 ± 0.1	86.5	1.4 ± 0.2	88.2	87.6	86.9	1.5 ± 0.2	1.1 ± 0.1	1.0 ± 0.2
		CT	87.7	1.1 ± 0.1	86.1	1.4 ± 0.2	86.6	87.8	87.1	1.4 ± 0.1	1.1 ± 0.2	1.0 ± 0.1
		TT	88.4	1.2 ± 0.2	88.4	1.5 ± 0.1	87.6	87.9	87.2	1.4 ± 0.1	1.2 ± 0.2	1.0 ± 0.1
	rs399579080	TT	88.4	1.1 ± 0.1	87.6	1.5 ± 0.1	86.8	88.2	87.5	1.5 ± 0.2	1.1 ± 0.1	1.1 ± 0.1
		TC	88.4	1.1 ± 0.2	86.7	1.4 ± 0.2	87.6	88.3	86.7	1.4 ± 0.1	1.1 ± 0.2	1.1 ± 0.1
		CC	88.5	1.2 ± 0.1	86.7	1.4 ± 0.1	88.2	86.8	87.1	1.5 ± 0.2	1.1 ± 0.2	1.0 ± 0.1
	rs421019907	CC	88.4	1.2 ± 0.2	87.9	1.5 ± 0.1	87.2	87.9	86.8	1.4 ± 0.1	1.2 ± 0.2	1.0 ± 0.1
		CT	88.4	1.1 ± 0.2	86.6	1.4 ± 0.2	87.4	87.2	87.5	1.4 ± 0.2	1.1 ± 0.2	1.0 ± 0.2
		TT	88.5	1.1 ± 0.1	86.5	1.4 ± 0.2	88.0	88.2	86.9	1.4 ± 0.1	1.2 ± 0.1	1.0 ± 0.1
	rs589708049	AA	—	—	—	—	—	—	87.5	—	—	1.1 ± 0.1
		GA	—	—	—	—	—	—	86.7	—	—	1.1 ± 0.1
		GG	—	—	—	—	—	—	87.1	—	—	1.0 ± 0.2
*BMPR1B*	rs408447622	CC	89.2	1.2 ± 0.2	86.9	1.4 ± 0.1	88.1	88.0	88.1	1.4 ± 0.1	1.2 ± 0.1	1.1 ± 0.1
		CT	88.5	1.1 ± 0.1	87.4	1.5 ± 0.1	86.9	87.1	86.1	1.4 ± 0.2	1.2 ± 0.1	1.1 ± 0.2
		TT	87.6	1.2 ± 0.1	86.7	1.4 ± 01	87.6	88.2	87.2	1.4 ± 0.1	1.2 ± 0.1	1.0 ± 0.1
	rs427897187	CC	88.9	1.1 ± 0.1	86.0	1.4 ± 0.2	86.7	88.1	88.1	1.5 ± 0.1	1.1 ± 0.1	1.1 ± 0.1
		CT	88.2	1.2 ± 0.2	86.0	1.4 ± 0.2	88.6	87.8	85.9	1.4 ± 0.1	1.1 ± 0.1	1.0 ± 0.1
		TT	88.0	1.1 ± 0.2	89.0	1.5 ± 0.1	87.2	86.9	87.2	1.5 ± 0.2	1.2 ± 0.1	1.0 ± 0.1
	rs409518279	GG	89.1	1.2 ± 0.2	86.9	1.4 ± 0.1	88.4	88.2	87.3	1.5 ± 0.2	1.2 ± 0.1	1.1 ± 0.1
		GA	88.0	1.1 ± 0.1	87.1	1.5 ± 0.1	86.9	87.9	86.3	1.4 ± 0.2	1.2 ± 0.1	1.0 ± 0.1
		AA	88.0	1.2 ± 0.2	87.0	1.5 ± 0.1	87.3	87.2	87.7	1.5 ± 0.2	1.2 ± 0.1	1.0 ± 0.1

**TABLE 8:** Gene, SNPs ID, genotypes and DRIL in the Awassi (*n* = 100), Sarda (*n* = 100), Bovška (*n* = 100) and Istrian Pramenka (*n* = 100) expressed as LSmeans, and the number of lambing events (recorded in a whole year from June to June) in the Jezersko‐Solčavska (*n* = 100) breed sheep.

Gene	SNP ID	Genotype	DRIL				Number of lambing
			Sarda	Awassi	Bovška	Istrian Pramenka	Jezersko‐Solčavska^a^
*BMP15*	rs592773279	AAG	177 ± 6	173 ± 18	177 ± 10	179 ± 7	35
		AAG/del	176 ± 9	172 ± 15		176 ± 5	31
		del	173 ± 5	171 ± 12	174 ± 8	182 ± 10	34
	rs55628000	AA	—	173 ± 13	—	—	—
		AG	—	171 ± 9	—	—	—
		GG	—	174 ± 15	—	—	—
	rs587958054	CC	—	172 ± 14	—	—	—
		CT	—	176 ± 12	—	—	—
		TT	—	174 ± 10	—	—	—
*GDF9*	rs410123449	CC	175 ± 3	173 ± 12	—	—	33
		CT	175 ± 4	171 ± 9	—	—	36
		TT	178 ± 7	173 ± 11	—	—	31
	rs422644056	GG	175 ± 6	172 ± 13	177 ± 8	—	32
		GA	179 ± 5	173 ± 14	175 ± 7	—	35
		AA	174 ± 4	177 ± 12		—	33
	rs160076413	CC	177 ± 3	172 ± 12	179 ± 9	181 ± 11	37
		CT	176 ± 3	176 ± 16	177 ± 7	177 ± 7	29
		TT	171 ± 5	175 ± 18	174 ± 4	180 ± 5	34
	rs108736417	TT	—	—	—	180 ± 11	—
		TA	—	—	—	173 ± 10	—
		AA	—	—	—		—
	rs160076408	CC	175 ± 4	171 ± 15	176 ± 8	179 ± 6	37
		CT	176 ± 8	176 ± 11	180 ± 9	180 ± 5	32
		TT	173 ± 5	174 ± 13		185 ± 10	31
	rs193637058	CC	176 ± 4	171 ± 9	177 ± 8	179 ± 9	29
		CT	173 ± 1	176 ± 12	176 ± 10	181 ± 7	38
		TT	—	174 ± 10	171 ± 5		33
	rs399579080	TT	175 ± 7	174 ± 12	177 ± 7	179 ± 9	31
		TC	176 ± 3	170 ± 10	170 ± 5	181 ± 7	36
		CC	—	170 ± 17	171 ± 7		33
	rs421019907	CC	175 ± 10	173 ± 8	177 ± 7	179 ± 9	27
		CT	176 ± 4	170 ± 10	170 ± 5	181 ± 7	35
		TT	—	178 ± 7	171 ± 7		38
	rs589708049	AA	—	—	177 ± 8	180 ± 5	33
		GA	—	—	175 ± 4	177 ± 9	38
		GG	—	—	173 ± 6	175 ± 10	29
*BMPR1B*	rs408447622	CC	176 ± 4	174 ± 6	177 ± 8	179 ± 12	32
		CT	174 ± 9	169 ± 10	176 ± 4	182 ± 10	38
		TT	173 ± 3	172 ± 12	173 ± 6	173 ± 8	30
	rs427897187	CC	177 ± 5	177 ± 15	177 ± 6	178 ± 7	33
		CT	175 ± 4	170 ± 8	175 ± 7	181 ± 6	33
		TT	173 ± 5	173 ± 16	173 ± 8	178 ± 9	34
	rs409518279	GG	176 ± 7	171 ± 12	177 ± 6	179 ± 8	29
		GA	173 ± 6	176 ± 5	176 ± 10	179 ± 5	34
		AA	173 ± 8	179 ± 9	172 ± 6		37

^a^ For the Jezersko‐Solčavska breed, the rams remained with the ewes year‐round, so the interval between ram introduction and lambing could not be evaluated. Only the total annual number of births was measured.

## 4 Discussion

Identifying genes that influence prolificacy and reproductive efficiency is a key objective in genetic selection programmes aimed at enhancing productivity in sheep farming. Therefore, this study investigated the nucleotide sequences of the *BMP15*, *GDF9* and *BMPR1B* genes to identify polymorphisms that could affect lambing rate and litter size in different sheep breeds reared for various purposes across three Mediterranean countries. Considering little‐researched breeds with diverse productive aptitudes is a strategic choice, driven by the need to shed light on the potential components that can be exploited through conscious selection to maximise the available resources. Typically, prolificacy varies greatly between meat and dairy breeds, as dairy producers have traditionally been less interested in prolificacy and even believe that lambs born from twin births tend to excessively exploit the udder during the period they are kept with the mother and excessively deprive the mother, who during that period must care for more newborns. In contrast, meat breeds highly value prolificacy, as it is crucial for the economic viability and profitability of their operations. Increased lambing rates provide meat producers with a faster return on investment through the sale and slaughter of a greater volume of lambs. This enhanced productivity is essential for the financial success and sustainability of meat‐focused sheep farming.

The *BMP15* (or *FecX*) gene is recognised as a major gene affecting prolificacy in sheep (Abdoli et al. 2016). This gene, expressed primarily in the oocyte, encodes a bone morphogenetic protein that is part of the transforming growth factor‐beta superfamily, which includes many growth and cell transformation factors (Calvo et al. 2020; Hanrahan et al. 2004). The *BMP15* gene plays an important role in early follicle growth by regulating the proliferation and differentiation of granulosa cells in both mono‐and poly‐ovulatory animals (Moore and Shimasaki 2005). Over the years, several mutations related to ovulation rate have been identified in the *BMP15* gene sequence in different sheep breeds (Bodin et al. 2007; Demars et al. 2013; Galloway et al. 2000; Hanrahan et al. 2004; Mullen et al. 2013). Several nonconservative mutations affecting litter size have also been identified in Iranian sheep breeds (Amini et al. 2018; Zamani et al. 2015). These mutations are likely to affect transcription, splicing and mRNA transcription or translation, which can all impact gene expression and, consequently, phenotype (Abdoli et al. 2016). In the present study, only one SNP was found within the *BMP15* gene exon 1 sequence of Sarda and Slovenian breeds, whereas the Awassi ewes exhibited one SNP in exon 1 and two in exon 2, but none corresponding to those known to influence litter size and ovulation rate, as seen in other sheep breeds (Amini et al. 2018; Hanrahan et al. 2004). The examined ewes did not exhibit the variations typically found in the *BMP15* gene that are associated with improved lambing rate and litter size in sheep. This suggests that the lambing rate and litter size observed in these breeds may be influenced by other genes or factors related to animal management and environment. Although some of the animals used in the study had twin and triplet births, no significant association was found between the genetic variations identified in the *BMP15* gene and the examined reproductive parameters.

The *GDF9* gene is another major gene and encodes a protein that is a member of the transforming growth factor beta (TGF‐b) superfamily. Indeed, in the ovary, it has been shown that *GDF9* is expressed throughout the folliculogenesis process, promoting follicle development and granulosa cell proliferation in ruminants (Bodensteiner et al. 2000, 1999). In our study, the *GDF9* gene showed the highest number of mutations compared to the other two loci analysed. These mutations correspond to one SNP in exon 1 and a total of eight SNPs in exon 2, variously distributed in the observed breeds. Five of the polymorphisms are nucleotide substitutions that result in amino acid changes. In one of these SNPs (rs589708049, also known as G8) the mutant homozygous genotype was associated with the sterility phenotype in different sheep breeds (Hanrahan et al. 2004). Given the presence of nonconservative mutations at this locus, we expected that the breeds we analysed would exhibit greater variability and that at least those more oriented towards meat production, such as Awassi and Jezersko‐Solčavska, would offer a more decisive correspondence with the most prolific phenotypes. However, despite the presence of several twin and even triplet births in these two breeds, no significant association emerged between the polymorphisms detected at this locus and the lambing rate and litter size.

Surely, these amino acid changes do not modify the protein structure, so no effect on reproductive performance was found. Moreover, many of the variations we found had a low allele frequency in the enrolled ewes, suggesting that a larger number of animals involved could make the investigation more reliable. Consequently, we hypothesised, also for this gene, that the prolificacy recorded in our study could be due to other genetic factors or to farm management, as emerged also in other countries (Ji et al. 2023).

The *BMPR1B* gene, as it is known, encodes a member of the bone morphogenetic protein (BMP) receptor family of serine/threonine transmembrane kinases. Its ligands are BMPs, which are members of the transforming growth factor beta (TGF‐β) superfamily. BMPs are involved in endochondral bone formation and embryogenesis. Several years ago, a single point mutation in the intracellular domain of the *BMPR1B* gene, called *FecB* (rs418841713), has been associated with an increase in the ovulation rate and, consequently, *BMPR1B* was the first gene associated with sheep prolificacy (Mulsant et al. 2001; Souza et al. 2001; Wilson et al. 2001). Because of its effect on ovulation rate and litter size in Booroola Merino sheep, this gene was also called the ‘*Booroola gene*’ (Smith et al. 1996). This mutation has been found in several sheep breeds, and some of these have shown an association with ovulation rate and litter size (Chu et al. 2007; Davis et al. 2006; Fogarty 2009; Walkden‐Brown et al. 2009). However, in sheep of non‐Asian breeds, this gene has been shown to increase the litter size, but at the same time, a decrease in the birth weight and the vitality of the lambs was reported (Mulsant et al. 2001; Walkden‐Brown et al. 2009). In the present study, the *FecB* mutation was not found in any of the examined breeds, and despite the identification of three polymorphisms in the *BMPR1B* locus, no association was observed with the reproductive parameters evaluated. These results confirmed the findings of Gootwine et al. (2008) in the Awassi breed, where only ewes improved by crossbreeding with the Merino breed, and hence carriers of the *Booroola* gene, showed an increase in prolificacy (Gootwine et al. 2008). The relatively low variability of the *BMPR1B* locus and lack of association with reproductive performance in the Sarda and Slovenian breeds may be due to their history as local breeds with relative isolation from more cosmopolitan populations. This highlights the need to explore beyond the most extensively studied genes and investigate other genomic regions that could help explain the variation in fertility and prolificacy across sheep breeds with different production purposes.

Overall, we expected to find more associations between the genetic variations observed in the three examined genes and the lambing rates and litter sizes of the studied populations, given the incidence of twin and triplet births observed in many of the enrolled ewes. However, our findings indicate that these breeds, likely due to the selective breeding practices employed by their producers, the limited introgression from more prolific breeds or their distinct production orientations, do not harbour mutations in these genes that are linked to their reproductive performance.

The absence of significant association implies that the high litter size found in some individuals is likely due to other management factors or other genes. Recent reports have indicated that a polymorphism in the *GREM1* gene, which is an antagonist of *BPMs*, influences the prolificacy of Awassi sheep (Imran et al. 2021). Therefore, it could be hypothesised that the *GREM1* gene in these sheep may regulate prolificacy by modulating the expression of BPMs, but this theory requires further studies to be confirmed.

In most cases, when rams are introduced into a flock, approximately 50% of the responding ewes develop a normal corpus luteum (CL) that is maintained during the normal luteal phase period, resulting in an oestrus ovulation around days 18–19 (Rosa and Bryant 2002). However, the remaining 50% develop a subnormal CL with a short lifespan that regresses around day 7, leading to a short cycle and a second ‘silent’ ovulation. This is followed by a CL that persists normally and results in a second oestrus cluster around day 24 of ram introduction (Mura et al. 2014).

Fecundity genes play a crucial role in regulating the proliferation and differentiation of granulosa and theca cells during folliculogenesis. In the early stages, these genes control the proliferation of granulosa cells (Fabre et al. 2006; Moore and Shimasaki 2005). Later, they influence the differentiating effects of FSH on follicular cells. Importantly, in large gonadotropin‐dependent antral follicles, *Fec*‐genes regulate follicular cell differentiation and exert a negative influence on FSH‐ and LH‐dependent progesterone production (McNatty et al. 2003). This suggests that they may be involved in delaying the luteinisation process in follicular cells prior to ovulation and the luteinisation triggered by the preovulatory gonadotropin surge (Fabre et al. 2006).

Given the involvement of these genes in follicular maturation, we expected to find associations between the studied polymorphisms and the distance in days from ram introduction to lambing. However, no statistical differences were observed for this parameter, suggesting that the individual variations in the DRIL trait are likely due to other factors not related to the genetic variations examined here.

The lack of significant associations observed in the present study between the genetic variations examined and the reproductive parameters analysed, considering the local nature of the studied breeds, could be due to the fact that these animals are well adapted to their territory of origin, and therefore, local environmental factors and management practices may play a more important role in determining their reproductive performance than the specific genetic variations analysed.

## 5 Conclusions

In conclusion, this study investigated the association between ovine prolificacy‐related mutations within the *BMP15*, *GDF9* and *BMPR1B* genes and reproductive performance in different sheep breeds reared for various production purposes in the Mediterranean basin. The results indicate that the DRIL, the lambing rate and the litter size observed in individual subjects are not associated with the genetic variations examined, suggesting that other genetic or environmental factors are involved. The choice to study local breeds, well‐adapted and integrated into their territory of origin, without admixture from more cosmopolitan breeds, leads us to consider the existence of more specific and understudied factors that may contribute to reproductive efficiency in these populations. Further investigation on loci still little investigated, such as the *GREM1* gene, an inhibitor of BMPs, could provide insights and potentially identify new markers useful for improving reproductive performance in Mediterranean sheep breeds. Additionally, future studies could benefit from two‐tailed sampling methods based on fertility records to increase the detection power of specific mutations associated with reproductive traits. This approach may enhance the understanding of genetic contributions to fertility and aid in identifying causative mutations in these populations.

## Author Contributions

All co‐authors have contributed equally to the present research. M.C.M., G.C., V.C. and S.L.: Conceptualisation; M.C.M., G.C., M.O., C.H., J.S., M.N., V.C. and S.L.: Data curation; G.C., M.O. and M.N.: Formal analysis; M.C.M., V.C. and S.L.: Funding acquisition; M.C.M., G.C., M.O., C.H., J.S., M.N., V.C. and S.L.: Investigation; M.C.M., G.C., M.O., C.H., J.S., M.N., V.C. and S.L.: Methodology; M.C.M., V.C. and S.L.: Project administration; M.C.M., G.C., M.O., C.H., J.S., M.N., V.C. and S.L.: Resources; G.C., M.O., J.S., M.N. and S.L.: Software; M.C.M., C.H., J.S., V.C. and S.L.: Supervision; M.C.M., C.H., J.S., V.C. and S.L.: Validation; M.C.M., G.C., M.O., C.H., J.S., M.N., V.C. and S.L.: Visualisation; M.C.M., G.C., V.C. and S.L.: Roles/Writing original draft; M.C.M., G.C., M.O., C.H., J.S., M.N., V.C. and S.L.: Writing, review and editing as well as article preparation. All co‐authors have approved the final draft of this article.

## Conflicts of Interest

The authors declare no conflicts of interest.

## Data Availability

The data that support the findings of this study are available from the corresponding author upon reasonable request.
